# Medullary Thyroid Cancer Risk and Mortality in Carriers of Incidentally Identified MEN2A *RET* Variants

**DOI:** 10.1001/jamanetworkopen.2025.17937

**Published:** 2025-06-27

**Authors:** Courtney E. West, Uyenlinh L. Mirshahi, Katherine S. Ruth, Luke N. Sharp, Ankit M. Arni, Clare Turnbull, Caroline F. Wright, Bijay Vaidya, Martina M. Owens, David J. Carey, Kashyap A. Patel

**Affiliations:** 1 Department of Clinical and Biomedical Sciences, University of Exeter Medical School, Exeter, United Kingdom; 2 Department of Genomic Health, Geisinger, Danville, Pennsylvania; 3 Institute of Cancer Research, London, United Kingdom; 4 Department of Endocrinology, The Royal Devon University Healthcare NHS Foundation Trust, Exeter, United Kingdom; 5 The Exeter Genomics Laboratory, The Royal Devon University Healthcare NHS Foundation Trust, Exeter, United Kingdom

## Abstract

**Importance:**

*RET* germline pathogenic variants cause multiple endocrine neoplasia type 2 (MEN2), which is associated with medullary thyroid cancer. With increasing incidental identification of these variants in asymptomatic individuals outside family screening, these individuals’ risk of medullary thyroid cancer and all-cause mortality without intervention remain unknown in this context.

**Objective:**

To evaluate the risk of medullary thyroid cancer and all-cause mortality in clinically unselected individuals with incidentally identified *RET* variants and assess whether the risk of medullary thyroid cancer differs from those with clinically ascertained *RET* variants.

**Design, Setting, and Participants:**

This prospective cohort study of 383 914 unrelated individuals from the clinically unselected UK population (UK Biobank, recruited in 2006-2010, with follow-up to June 2023) and 122 640 unrelated individuals from a US health system (Geisinger MyCode cohort, recruited 2004-2020, with follow-up to October 2023) compared medullary thyroid cancer risk in these cohorts with 1078 individuals who were clinically ascertained with suspicion of MEN2 from a UK routine practice.

**Exposures:**

*RET* germline pathogenic variants causing MEN2.

**Main Outcomes and Measures:**

Frequency and the spectrum of pathogenic *RET* variants, risk of clinically present medullary thyroid cancer, and all-cause mortality without thyroidectomy were assessed using proportions with exact binomial 95% CIs and survival analysis adjusted for age at recruitment and sex.

**Results:**

In the UK Biobank, 169 unrelated individuals (mean [SD] age at recruitment, 57.0 [8.1] years; 94 male [55.6%]) had a pathogenic *RET* variant (prevalence, 0.04% [95% CI, 0.04%-0.05%]). In the US health system–based cohort, 77 unrelated individuals (mean [SD] age at recruitment, 56.2 [17.8] years; 45 female [58.4%]) had a pathogenic *RET* variant (prevalence, 0.06% [95% CI, 0.05%-0.78%]). The variants were predominantly from the moderate-risk category per American Thyroid Association guidelines (168 individuals [99.4%] and 75 individuals [94.8%], respectively). The Kaplan-Meier estimated medullary thyroid cancer risk by age 75 years in variant carriers in the UK population was 2.2% (95% CI, 0.7%-6.9) and 19.3% (95% CI, 6.4%-30.2%) in US health system cohort. These risks were significantly lower compared with the clinically ascertained cohort with the matched variants (95.7% [95% CI, 82.1%-99.7%]). In the UK Biobank, most variant carriers (166 [98.2%]) did not undergo thyroidectomy, and their all-cause mortality by age 75 years was similar to noncarriers (6.1% [95% CI, 2.7%-13.8%] vs 5.7% [95% CI, 5.6%-5.8%]), with consistent findings in the US health system cohort.

**Conclusions and Relevance:**

In this cohort study, moderate-risk *RET* variants were most common in incidental cases. The variants were associated with a substantially lower medullary thyroid cancer risk than clinically ascertained cases. This evidence addresses a current knowledge gap, enabling more informed clinical decision-making.

## Key Points

**Question:**

What are the medullary thyroid cancer risks for individuals with incidentally identified *RET* variants, and do they differ from individuals with *RET* variants ascertained clinically?

**Findings:**

In this cohort study of 506 544 unrelated individuals, incidentally identified moderate-risk *RET* variants were associated with substantially lower medullary thyroid cancer risk compared with clinically ascertained cases with suspected multiple endocrine neoplasia type 2 (2.8%-19.0% vs 98.2%). All-cause mortality among carriers with incidentally detected *RET* variants was similar to that of noncarriers (6.2% vs 5.7%).

**Meaning:**

These findings suggest that incidentally discovered moderate-risk *RET* variants are associated with markedly lower cancer risk, providing crucial information to guide treatment decisions and address an important knowledge gap in genetic risk assessment.

## Teaser

This cohort study evaluates medullary thyroid cancer risk and mortality in individuals with incidentally identified multiple endocrine neoplasia type 2A (MEN2A) *RET* variants and these differ from clinically ascertained cases.

## Introduction

Gain-of-function pathogenic variants in the *RET* oncogene cause multiple endocrine neoplasia type 2 (MEN2), an aggressive autosomal-dominant endocrine cancer syndrome.^1,2^ Medullary thyroid cancer is the most common feature, affecting 95% to 100% of patients with MEN2.^1,2^ Genetic testing of *RET* is widely recommended for all patients with medullary thyroid cancer and/or suspected MEN2.^1^ If a patient is found to have a pathogenic *RET* variant, cascade testing of their relatives is performed, and heterozygous relatives are considered for early curative prophylactic total thyroidectomy due to a high risk of medullary thyroid cancer.^2,3,4^

Current guidelines recommend reporting incidentally identified pathogenic *RET* variants in asymptomatic individuals.^5^ In these individuals, a pathogenic *RET* variant is identified as part of secondary findings outside the family screening. This identification is made via diagnostic clinical exome and/or genome sequencing for unrelated indications or as part of research studies (clinically unselected cases). Since 2013, when the American College of Medical Genetics and Genomics first recommended this approach for MEN2A-causing *RET* variants,^5^ it has been widely emulated, including by the UK’s 100 000 Genomes Project, European Society for Medical Oncology,^6^ and recent newborn genomic screening proposals.^7,8^ The presumption of net clinical benefit that underpins these recommendations has been extrapolated from the benefit-risk balance ascribed to clinically identified families based on the lack of data in incidentally detected cases. This difference in approach warrants careful evaluation, particularly given the distinct contexts in which these variants are being identified. Recent studies have shown that analyses of clinically ascertained families tend to overestimate the risk for monogenic disorders compared with those whose pathogenic variants were identified through incidental findings.^9,10^ Consequently, determining the frequency, variant spectrum, and rate of clinical medullary thyroid cancer presentation in incidental *RET* variant carriers, as well as understanding how these differ from clinically ascertained cohorts, is important to informing the current recommendations for incidentally identified cases. This knowledge gap has become increasingly pressing with the widespread adoption of exome and genome sequencing in clinical and research settings, coupled with recommendations to report incidental *RET* findings, highlighting the urgent need to better understand medullary thyroid cancer risk in incidental cases.

We therefore aimed to identify the frequency of pathogenic *RET* variants, the spectrum of variants, and their associated risks with medullary thyroid cancer and all-cause mortality without intervention in clinically unselected individuals. We analyzed detailed genetic and clinical data from more than 500 000 clinically unselected individuals who underwent genomic sequencing. We also compared the risk of clinically presented medullary thyroid cancer in these individuals with that of individuals with clinically ascertained variants to highlight the differences between these 2 groups.

## Methods

### Study Populations

This cohort study included 3 study populations: the UK Biobank cohort, Geisinger MyCode cohort, and Exeter clinical cohort. The UK Biobank Research Ethics Committee approved the study, with all participants providing written informed consent.^11^ The Geisinger Institutional Review Board determined the study to not be human participants research, and participants consented to share their routinely collected electronic health record information for research, including clinical diagnoses, procedures, cancer registry data, medications, and laboratory results. Exeter patients or parents provided informed consent prior to genetic testing, and part of the data from this cohort was previously published.^12^ The study followed the Strengthening the Reporting of Observational Studies in Epidemiology (STROBE) reporting guideline.

#### UK Biobank

The UK Biobank is a large UK population cohort of 500 000 clinically unselected individuals of African, European, South Asian, and other or unknown ancestry (ie, any ancestry other than African, European, or South Asian) recruited between 2006 and 2010 at age 40 to 69 years.^13^ It contains phenotypic data provided through self-report questionnaires, hospital records, cancer and death registries, and linked general practitioner records available both at baseline and follow-up.^13^ We used 383 914 individuals from an exome sequencing data release on 450 000 individuals who were unrelated (up to third degree).^14^ We used unrelated individuals to prevent undue influence of large families on variant frequency in the cohort (15.6% related). We used linked data on phenotypes at baseline and follow-up (up to October 31, 2022). Baseline characteristics of this cohort are summarized in eTable 1 in Supplement 1.

#### Geisinger MyCode Cohort

Geisinger MyCode is a health system–based cohort from the US consisting of 340 423 unselected individuals of African, European, South Asian, and other or unknown ancestry (ie, any ancestry other than African, European, or South Asian) who sought care at Geisinger, a health care system in central and northeastern Pennsylvania. The cancer registry included histologic data of cancers diagnosed and treated at Geisinger since 1943. We used a subset of 122 640 unrelated (up to third degree) MyCode participants with exome sequencing, generated as part of the DiscovEHR collaboration between Geisinger and the Regeneron Genetics Center.^15^ We used unrelated individuals to prevent undue influence of large families on variant frequency in the cohort (36% related up to third degree). We used data at baseline (the date of exome sequencing) and follow-up (up to June 1, 2023). The cohort characteristics at completion of exome sequencing (baseline) are summarized in eTable 1 in Supplement 1 and have been extensively described.^9^

#### Exeter Clinical Cohort

The Exeter clinical cohort included 1078 unrelated individuals (probands) from routine clinical practice in the UK with suspected MEN2 who were referred to the Exeter Genomics Laboratory for *RET* gene sequencing from 1995 to 2018. We did not have ancestry information for this cohort. Of these individuals, 117 were found to have a heterozygous germline pathogenic activating *RET* variant. The baseline characteristics of the clinical cohort are summarized in eTable 1 in Supplement 1.

### Genetic Analysis

We used exome sequencing data released centrally by the UK Biobank. The detailed process of exome sequencing and sample and variant filtering have been described by Szustakowski et al^13^ and are publicly available.^16^

For the Geisinger MyCode cohort, we used exome sequencing performed as part of the DiscovEHR collaboration between Geisinger and the Regeneron Genetics Center.^12^ The detailed method for exome sequencing and sample and variant filtering has been described previously by Mirshahi et al.^9^

For the Exeter clinical cohort, we used Sanger sequencing or targeted gene panels for clinical *RET* sequencing. Sequencing coverage included but was not limited to exons 10, 11, 13, 14, and 16 of *RET*.^12^ Clinical scientists at the Exeter Genomics Laboratory, the Royal Devon and Exeter Hospital, analyzed the variants as part of routine diagnostic care. This laboratory has provided *RET* genetic testing for the UK population since 1995 and is considered a center of excellence for *RET* genetic testing in the UK.

### Variant Classifications in Clinically Unselected Cohorts

We annotated *RET* variants using MANE Select transcript NM_020975.5. We reviewed all heterozygous missense variants in *RET* across the 2 clinically unselected cohorts. Variants were classified as pathogenic if previously reported in probands with MEN2 and deemed likely pathogenic or pathogenic according to American College of Medical Genetics and Genomics and Association for Molecular Pathology guidelines^5^ in conjunction with the Association for Clinical Genomic Science best practice guidelines for variant classification in rare disease.^17^ We used 3 *RET* pathogenic variant databases and the ClinVar database to check for previous reports of the variants in patients with MEN2.^12,18,19,20^ We manually reviewed the sequence reads supporting pathogenic variants using the Integrative Genomics Viewer, assessing read depth and allele fraction to remove false-positive results or somatic mosaics. All pathogenic variants were considered high quality based on 2 independent reviews (U.L.M. and K.A.P.). We also classified variants into highest-, high-, or moderate-risk categories based on American Thyroid Association recommendations.^3^

### Phenotype Definitions

#### Clinically Unselected Cohorts

We used 2 definitions to identify clinically presented medullary thyroid cancer cases. Our main definition included thyroid cancer with medullary histology, while our broad definition additionally included any thyroid cancer or thyroidectomy (partial, subtotal, and total) regardless of indication. This broader definition captured individuals without cancer registry data, with missing histology, or who underwent prophylactic surgery. We identified cases using hospital episodes statistic (*International Statistical Classification of Diseases, Tenth Revision*), operation record (Office of Population Censuses and Surveys), cancer registry, death registry, general practitioner–recorded, and self-reported data. The main definition served as our primary analysis, with the broad definition used for sensitivity analysis. Data sources and codes for both definitions are detailed in eTable 2 in Supplement 1.

We also identified individuals with potential pheochromocytoma, which is a recognized, but less frequent feature of MEN2.^21^ Due to the rarity of pheochromocytoma, we used a broad definition that included histologically confirmed pheochromocytoma or any adrenal tumor or adrenal surgery to reduce the chance of missing any cases. Data sources and codes for both definitions are detailed eTable 2 in Supplement 1.

All data were collected up to the date of recruitment (UK Biobank) and follow-up to October 2022. For the Geisinger MyCode cohort, we used data up to the date of exome sequencing. The follow-up data excluded individuals whose incidental findings of pathogenic *RET* variants were reported back to them as part of the MyCode Genome Screening and Counseling Program, which discloses results to biobank participants. The findings for these individuals were recently published elsewhere.^22^ For individuals for whom incidental findings have not yet been reported, we presented follow-up data from exome sequencing to June 2023. We used death registration to assess all-cause mortality from recruitment in all clinically unselected cohorts.

#### Exeter Clinical Cohort

The referring clinician reported the presence or absence of medullary thyroid cancer and age at medullary thyroid cancer diagnosis at the time of referral to the Exeter Genomics Laboratory for genetic testing. If not explicitly stated, the age at diagnosis was taken as the age of referral for analysis of the *RET* gene.

### Statistical Analysis

We assessed penetrance by calculating the proportion of medullary thyroid cancer cases among carriers with *RET* pathogenic variants. We used exact binomial 95% CIs for the proportions. Age-related penetrance was estimated using Kaplan-Meier survival analysis, given the availability of age at diagnosis and surgery information. Participants were followed up until study end (October 2022 for UK Biobank and June 2023 for Geisinger MyCode), censoring at disease onset (using the main or broad definition), or death. We analyzed medullary thyroid cancer incidence rates and all-cause mortality from recruitment to study end in both cohorts. Log-rank tests for equality were performed to compare age-dependent penetrance between groups, and Cox proportional hazard ratios (HRs) were computed, adjusting for age at recruitment and sex. The log-rank test *P* value comparing 2 curves was a 2-sided test, with *P* < .05 considered significant. We used Stata, version 16 (StataCorp LLC); the survminer package in R, version 0.4.9 (R Foundation for Statistical Computing); and Scipy and statsmodels in Python, version 3.9.18 (Python Software Foundation) for the analyses and the matplotlib and seaborn packages in Python for plotting.

## Results

### Prevalence of MEN2-Causing Pathogenic *RET* Variants in Clinically Unselected Cohorts

In the analysis of 383 914 unrelated individuals with exome data in the UK Biobank, 169 (mean [SD] age at recruitment, 57.0 [8.1] years; 75 female [44.4%] and 94 male [55.6%]; 2 of African [1.2%], 153 European [90.5%], 2 South Asian [1.2%], and 12 of other or unknown ancestry [7.1%]) were identified as carriers of 1 of 17 different MEN2-causing pathogenic *RET* variants (prevalence of 1 in 2500; 0.044% [95% CI, 0.038%-0.051%]) (Table). Of these 17 variants, 16 (94%) were moderate risk (based on American Thyroid Association guidelines) and present in 168 individuals (99.4%), and 1 individual (0.6%) carried a high-risk variant (Table; eTable 3 in Supplement 1). The most common variant was p.Val804Met, found in 95 individuals (56.2%) (Table).

**Table.** Characteristics of Participants With a Pathogenic Medullary Thyroid Cancer–Causing *RET* Variant Across Study Cohorts

Characteristic	Population cohort, UK Biobank (unrelated)^a^	US health care system cohort, Geisinger MyCode (unrelated)	Clinical cohort, Exeter, UK
All *RET* pathogenic variant carriers, No. of total cohort (%)	169 of 383 914 (0.04)	77 of 122 640 (0.06)	117 of 1078 (10.85)
Age at recruitment, mean (SD), y	57.0 (8.1)	56.2 (17.8)	47.3 (18.0)
Sex, No. (%)			
Female	75 (44.4)	45 (58.4)	75 (64.1)
Male	94 (55.6)	32 (41.6)	42 (35.9)
Ancestry			
Admixed American	0	1 (1.3)	NA
African	2 (1.2)	1 (1.3)	NA
European	153 (90.5)	74 (96.1)	NA
South Asian	2 (1.2)	0	NA
Other or unknown^b^	12 (7.1)	1 (1.3)	NA
Pathogenic variant classification^c^			
Highest risk			
p.Met918Thr	0	0	18 (15.5)
High risk			
p.Cys634Arg/Phe/Trp/Tyr or p.Ala883Phe	1 (0.6)	4 (5.2)	35 (30.2)
Moderate risk			
All	168 (99.4)	73 (94.8)	64 (54.7)
p.Gly533Cys	NA	NA	2 (1.7)
p.Cys609Phe/Tyr or p.Cys620Gly/Arg/Ser/Phe/Tyr/Trp or p.Asp631Tyr^d^	8 (4.7)	8 (10.4)	17 (14.5)
p.Cys611Arg	0	0	1 (0.9)
p.Cys618Gly/Arg/Ser	0	0	12 (10.3)
p.Lys666Asn	14 (8.3)	6 (7.8)	0
p.Lys666Glu	6 (3.6)	2 (2.6)	2 (1.7)
p.Glu673Ala	0	0	1 (0.9)
p.Glu768Asp, p.Ser904Phe, or p.Met918Val	7 (4.1)	2 (2.6)	0
p.Leu790Phe	14 (8.3)	0	7 (6.0)
p.Val804Met	95 (56.2)	30 (39.0)	14 (12.0)
p.Val804Leu	15 (8.9)	0	0
p.Ser891Ala	9 (5.3)	25 (32.5)	8 (6.8)

Abbreviation: NA, not available.

^a^ Variants with fewer than 5 carriers were combined in line with UK Biobank policy.

^b^ Other included all ancestries not listed in the table.

^c^ Pathogenic variants grouped according to American Thyroid Association guidelines.^3^

^d^ Extracellular moderate-risk variants.

The prevalence of pathogenic *RET* variants in the large US health system–based Geisinger MyCode cohort (77 carriers of 122 640 cohort participants; mean [SD] age at recruitment, 56.2 [17.8] years, 45 female [58.4%] and 32 male [41.6%]; 1 each of Admixed American, African, and other or unknown ancestry [1.3%] and 74 of European ancestry [96.1%]) was slightly higher at 1 in 1666 (0.06% [95% CI, 0.05%-0.78%]). This prevalence may have been attributable to the hospital-based setting, which would have included relatively more individuals with self-presented MEN2. Aligning with the UK Biobank findings, 71.4% (10 of 14) of variants were moderate risk and present in 75 individuals (94.8%), with p.Val804Met being the most common and observed in 30 *RET *carriers (39.0%), while 28.6% (4 of 10 variants) were high risk observed in 4 carriers (5.2%) (Table). The frequency of pathogenic variants across ancestry in these 2 cohorts was similar (eTable 4 in Supplement 1).

### Medullary Thyroid Cancer Risk in *RET* Pathogenic Variant Carriers 

#### UK Biobank

**Figure 1.**
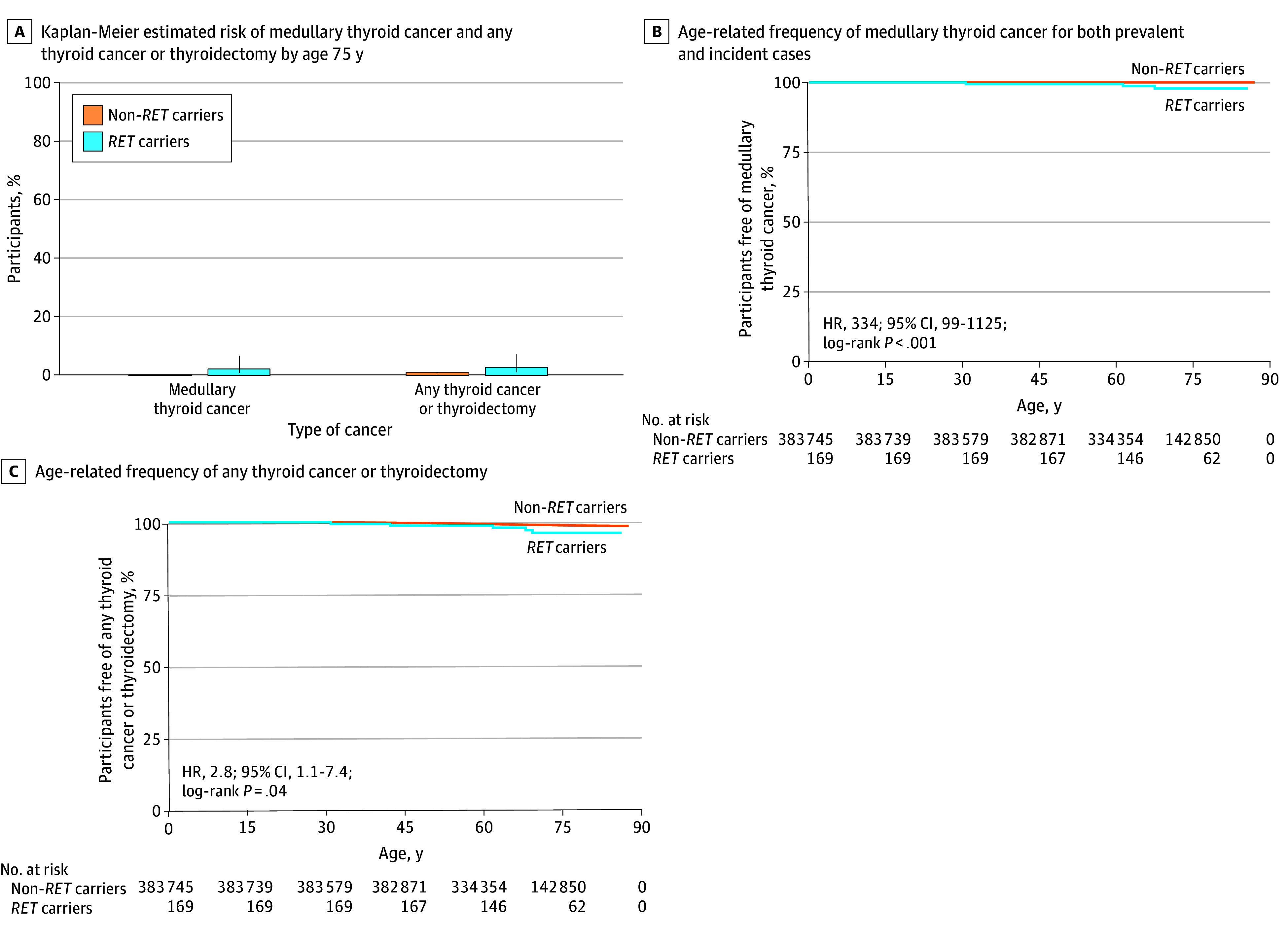
The Age-Related Risk of Medullary Thyroid Cancer in Individuals With Pathogenic *RET* Variants in a Clinically Unselected UK Biobank Cohort A. Any thyroidectomy included partial, total, or subtotal for any indication by *RET* pathogenic variant status (n = 169 carriers and 383 745 noncarriers). The error bars represent 95% CIs. B and C. Log-rank tests showed significant differences between *RET* carriers and noncarriers (*P* < .001 for medullary thyroid cancer; *P* = .02 for broad definition).

Among 169 *RET* pathogenic variant carriers in UK Biobank, only 2 (1.2% [95% CI, 0.1%-4.2%]) had medullary thyroid cancer at recruitment, 1 with a high-risk variant and 1 with a moderate-risk variant (eTables 5 and 6 in Supplement 1). After 2299 person-years of follow-up (median follow-up, 13.8 [IQR, 13.0-14.4] years) at a mean (SD) age of 70.6 (8.1) years, we identified 1 additional case (0.41 [95% CI, 0.11-2.35] cases per 1000 person-years). Thus, the observed frequency of medullary thyroid cancer was 1.8% (3 of 169 participants) in the UK Biobank, with a Kaplan-Meier estimated risk of medullary thyroid cancer by age 75 years of 2.2% (95% CI, 0.7%-6.8%) (Figure 1A and B). Although the absolute risk was low, it was still higher in *RET* carriers compared with noncarriers (age- and sex-adjusted HR, 334 [95% CI, 99-1125]; *P* < .001). Use of a broader definition that included any thyroid cancer or thyroidectomy identified 2 additional cases for an observed frequency of 2.9% (5 of 169 carriers) and a Kaplan-Meier–estimated risk increase to 2.8% (95% CI, 1.0%-7.4%) by age 75 years (Figure 1A and C; eTable 6 in Supplement 1). In line with the broader, less-specific definition, the HR was lower (2.8 [95% CI, 1.1-7.6]; *P* = .04). All 3 additional cases had thyroidectomy for non–medullary thyroid cancer indications (eTable 5 in Supplement 1). The penetrance was similar between p.Val804Met and other moderate-risk variants (1.12% [95% CI, 0.02%-5.66%] vs 1.43% [95% CI, 0.03%-7.42%]) (Fisher exact P > .99), as well as between intracellular and extracellular moderate-risk variants (eTable 6 in Supplement 1). The results by ancestry and by variant risk categories are provided in eTables 4 and 6 in Supplement 1, respectively.

#### US Health System Cohort

**Figure 2.**
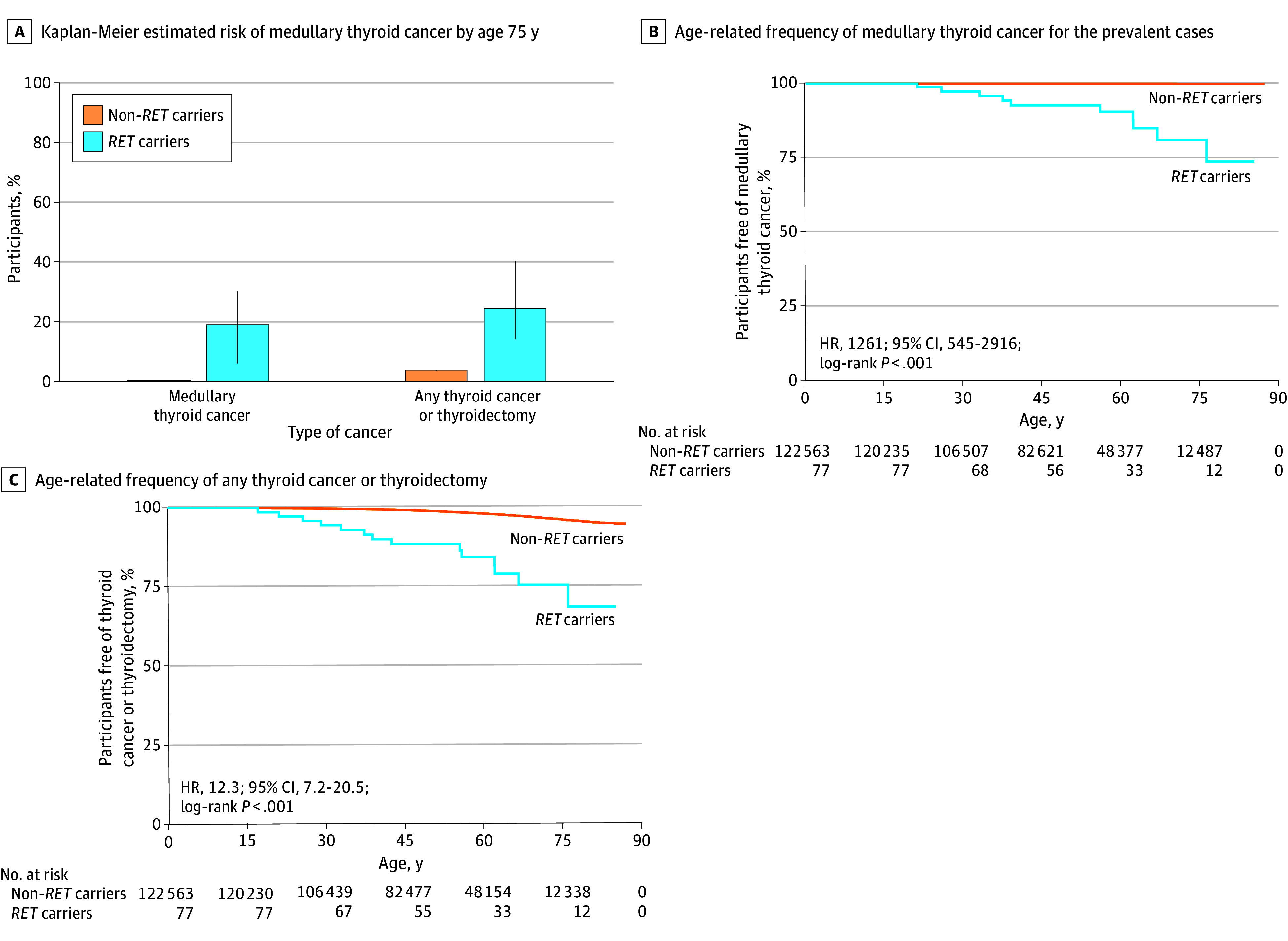
Risk of Medullary Thyroid Cancer in Clinically Unselected Individuals With Pathogenic *RET* Variants Replicated in Health System–Based Cohort A. Any thyroidectomy included partial, total, or subtotal for any indication by *RET* pathogenic variant status (n = 77 carriers and 122 563 noncarriers). The error bars represent 95% CIs. B and C. Log-rank test showed significant differences between *RET* carriers and noncarriers (*P* < .001 for medullary thyroid cancer and for broad definition).

To validate our findings, we analyzed 122 640 unrelated individuals from the US-based Geisinger MyCode cohort. Among 77 *RET* pathogenic variant carriers (52 [67.5%] of whom underwent Sanger sequencing as part of the MyCode Genome Screening and Counseling Program to confirm the variant), 10 were found to have medullary thyroid cancer. This finding provided an observed frequency of medullary thyroid cancer of 13.0% in our cohort, with the Kaplan-Meier–estimated risk of medullary thyroid cancer by age 75 years being 19.0% (95% CI, 6.4%-30.2%) (Figure 2A and B). The observed frequency of the broad definition was 24.6% (19 participants) with the Kaplan-Meier–estimated risk of medullary thyroid cancer by age 75 years being 24.3% (95% CI, 10.7%-35.9%) (Figure 2A and C). Medullary thyroid cancer penetrance was 25.0% (95% CI, 0.6%-80.5%) in high-risk *RET* variant carriers (1 of 4) and 12.3% (95% CI, 5.8%-22.1%) in moderate-risk carriers (9 of 73 carriers) (eTables 5 and 6 in Supplement 1). The overall age- and sex-adjusted HRs for medullary thyroid cancer in all variant carriers was 1261 (95% CI, 545-2916) and for the broad definition, 12.3 (95% CI, 7.2-20.5) (both *P* < .001). Meta-analysis across the 2 cohorts for the risk of medullary thyroid cancer refined the precision of the HRs to 480 (95% CI, 9-951) (eFigure 1 in Supplement 1). Of 77 participants with medullary thyroid cancer in Geisinger MyCode, 28 (36.4%) were yet uninformed of their *RET* mutation status as part of the MyCode Genome Screening and Counseling Program, and none of them developed medullary thyroid cancer over 538 person-years of follow-up (median, 7.0 [IQR, 4.4-8.9] years) at a median age of 74.5 years (IQR, 58.1-84.3 years). Outcomes for the remaining 49 participants informed through the MyCode Genome Screening and Counseling Program were recently published.^22^ Medullary thyroid cancer cases stratified by American Thyroid Association risk categories and the common p.Val804Met variant are detailed in eTable 6 in Supplement 1.

### Pheochromocytoma Risks in *RET* Pathogenic Variant Carriers From the UK Biobank and US Health System Cohorts

Along with medullary thyroid cancer, MEN2 causes pheochromocytoma, although this is less common, but screening is recommended.^21^ We therefore assessed the risk of pheochromocytoma in *RET* carriers from clinically unselected cohorts. Of the 169 carriers in the UK Biobank, only 1 reported having pheochromocytoma (a carrier of the high-risk variant and medullary thyroid cancer). In the Geisinger MyCode cohort, 2 of the 77 *RET* variant carriers had potential pheochromocytoma. Both harbored a high-risk variant and had medullary thyroid cancer. This lower penetrance is consistent with our observation of lower medullary thyroid cancer risk in these individuals.

### All-Cause Mortality in Untreated *RET* Variant Carriers

**Figure 3.**
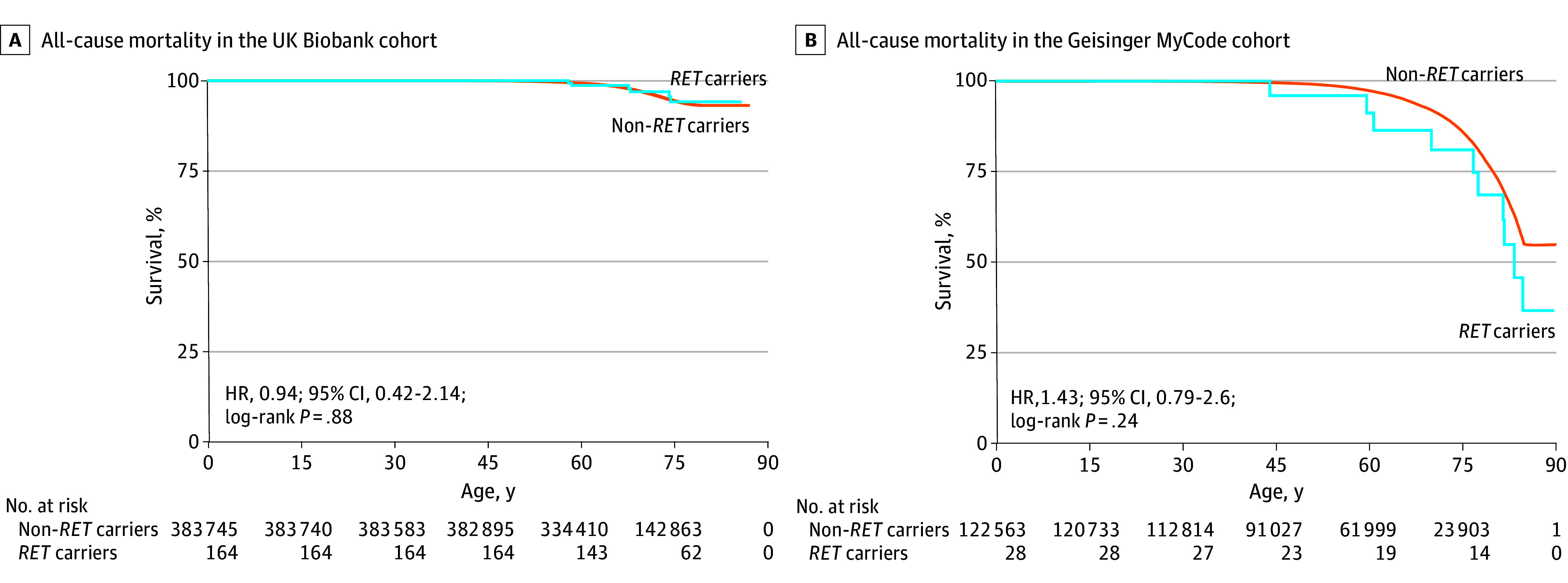
Pathogenic *RET* Variant Carrier and Noncarrier All-Cause Mortality Without Prophylactic Thyroidectomy

A genotype-first approach uniquely enabled us to assess mortality outcomes in untreated *RET* variant carriers, previously impossible as withholding intervention was considered unethical, particularly in high-risk family members. In the UK Biobank, 166 of the 169 (98.2%) variant carriers had not undergone thyroidectomy. Over 2299 person-years of follow-up (median, 13.8 [IQR, 13.0-14.4] years), their all-cause mortality was comparable to noncarriers (6.1% [95% CI, 2.7%-13.8%] vs 5.7% [95% CI 5.6%-5.8%] by age 75 years) (log-rank *P* = .79), with an HR of 0.94 (95% CI, 0.42-2.14; *P* = .88) (Figure 3A). Mortality rates were similar between carriers with and without thyroidectomy, though the analysis was limited by few thyroidectomy cases (0 of 5 vs 6 of 164; Fisher exact test *P* > .99). These findings were consistent in the Geisinger MyCode cohort, in which 0 of the 28 uninformed *RET* variant carriers had undergone thyroidectomy over 140 person-years of follow-up (median, 5.0 [IQR, 2.7-7.9] years) and showed no excess mortality compared with noncarriers by age 75 years (11.6% [95% CI, 0.0%-21.8%] vs 13.9% [95% CI, 13.5%-14.2%]) (log-rank *P* = .50), with an HR of 1.43 (95% CI, 0.79-2.6; log-rank *P* = .24) (Figure 3B).

### Medullary Thyroid Cancer Penetrance in *RET* Variant Carriers vs Clinical Cases

**Figure 4.**
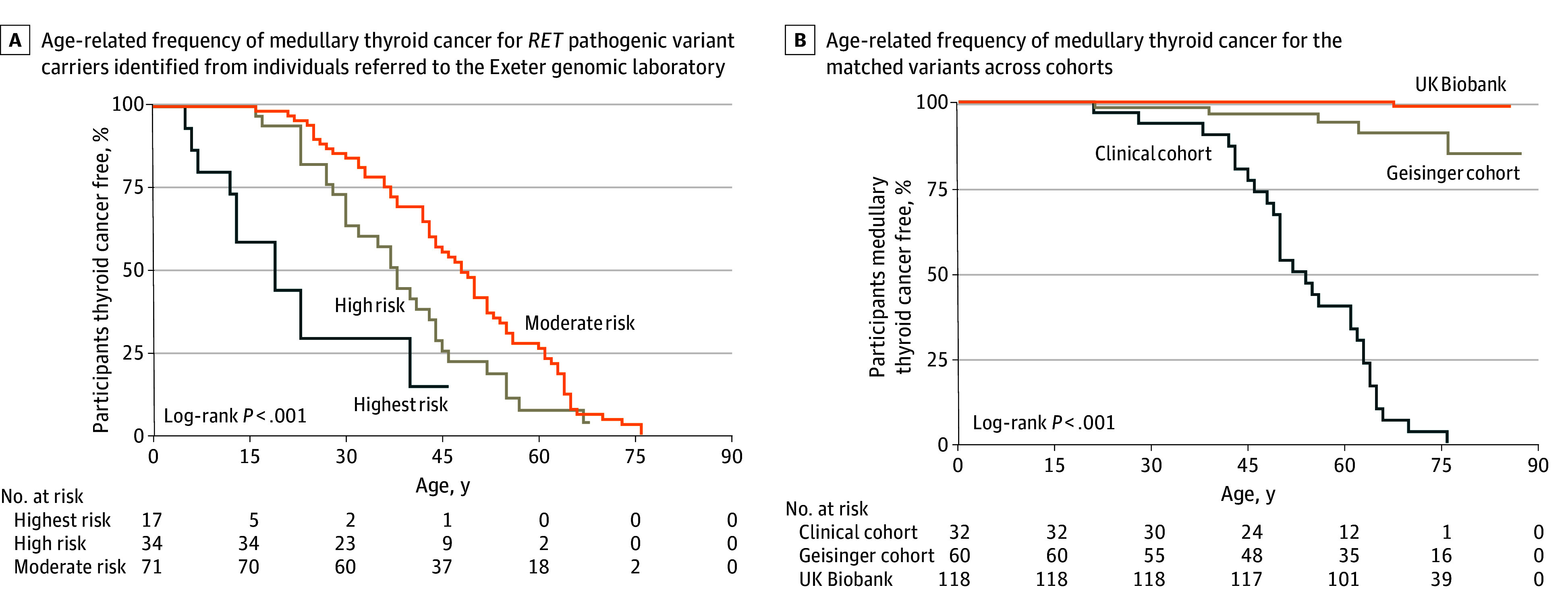
Age-Related Risk of Medullary Thyroid Cancer in Individuals With *RET* Pathogenic Variant Ascertained Clinically and in a Clinically Unselected Population and Health System–Based Cohort With the Matched Variants

We compared medullary thyroid cancer penetrance between clinically unselected cases and 1078 probands referred from UK routine clinical practice due to suspected MEN2. Of these clinical referrals, 117 (10.9%; mean [SD] age at recruitment, 47.3 [18.0] years; 75 female [64.1%] and 42 male [35.9%]) carried a pathogenic *RET* variant (64 [54.7%] with moderate risk, 35 [30.2%] with high risk, and 18 [15.5%] with highest risk) (Table). Age-dependent medullary thyroid cancer penetrance in clinical referrals was 68.6% [95% CI, 37.0%-94.5%] and 84.3% [95% CI, 51.3%-99.1%] at age 30 and 45 years for highest-risk variants, 35.3% [95% CI, 21.8%-53.8%] and 70.6% [95% CI, 54.5%-85.1%] for high-risk variants, and 16.0% [95% CI, 9.0%-27.9%] and 45.0% [95% CI, 33.5%-58.5%] for moderate-risk variants (Figure 4A). Analysis of only variants present across all 3 cohorts showed a significantly higher medullary thyroid cancer penetrance at age 75 years in clinically ascertained cases (95.7% [95% CI, 82.1%-99.7%]) compared with the Geisinger cohort (15.9% [95% CI, 8.0%-30.2%]) and UK Biobank cohort (1.3% [95% CI, 0.2%-8.7%]) (both *P* < .001) (Figure 4B). This difference persisted for the analysis of only the most common variant p.Val804Met (92.3% vs 1.6% vs 0%) and other moderate-risk variants (eFigure 2 in Supplement 1). Similarly, for high-risk variants, we observed directionally consistent results though limited by small numbers (2 of 5 carriers [40.0%] vs 29 of 35 carriers [82.9%]) (Fisher exact *P* = .03) (eFigure 2C and eTable 6 in Supplement 1).

## Discussion

Our cohort study of genomic screening of more than 500 000 unselected individuals from 2 different ascertainment settings suggests that incidental pathogenic activating *RET* variants occurred in approximately 1 in 2000 individuals, 30-fold more common than previously estimated.^23^ The majority were moderate-risk variants associated with low medullary thyroid cancer risk and no excess in all-cause mortality without thyroidectomy.

Accurate medullary thyroid cancer risk information is essential for effective risk-benefit discussions with individuals carrying incidentally identified pathogenic *RET* variants. While intervention choice remains personal, our study provides, to our knowledge, the first evidence in this context to support informed decision-making by health care professionals and genetic counselors. We present penetrance values for different genotype-first settings, which may enable genetic counselors to provide more appropriate risk estimates. We show that current risk estimates based on family studies are associated with an overestimated risk, a pattern now observed across several monogenic disorders, including breast cancer, Lynch syndrome, and diabetes.^9,10,24^ Our findings are primarily associated with moderate-risk variant carriers, as our sample size for high-risk variants was limited (5 individuals, 2 of whom with medullary thyroid cancer), highlighting the importance of variant-specific penetrance estimates, as deleterious variants within the same gene can impart different risks for the same condition. The consistency of results, with a broad disease definition and longitudinal data across 2 health care settings in 2 countries and similar results for pheochromocytoma, supports the validity of our results. Our p.Val804Met penetrance estimate aligns with that of Loveday et al,^25^ who estimated 4% (95% CI, 0.9%-8%) based on maximum tolerated allele frequency, as well as a lower estimate (3%-20%) observed from family-based studies.^26^ The risk estimates for moderate-risk variants excluding p.Val804Met were similar to other moderate-risk variants (eTable 5 in Supplement 1). Although extracellular moderate-risk variants compared with intracellular variants showed higher medullary thyroid cancer risk in Geisinger MyCode but not UK Biobank carriers (eTable 5 in Supplement 1), the higher penetrance in the Geisinger cohort may reflect its health care–based recruitment, which would have included relatively more individuals with self-presented MEN2. This finding is consistent with Geisinger’s higher medullary thyroid cancer prevalence (18.7 per 100 000 individuals) compared with previous population estimates (3.8 per 100 000 individuals). While UK Biobank’s medullary thyroid cancer prevalence (7.5 per 100 000 individuals) aligns with population estimates, its healthy volunteer effect suggests that these penetrance estimates may represent a lower bound of true population penetrance.^27,28^

Our results contrast with a recent study by Pichardo et al,^22^ which reported outcomes from 20 Geisinger MyCode participants undergoing prophylactic thyroidectomy after incidental detection of moderate-risk *RET* p.Ser891Ala (n = 14) and p.Val804Met (n = 6). Of 75 moderate-risk *RET* carriers offered surgery, only 20 (26.7%) proceeded, with 12 showing histologically confirmed medullary thyroid cancer, predominantly stage 1 disease (10 of 12 [83.3%]). Outcomes for individuals declining surgery were not reported. The absence of excess mortality and a lower rate of clinically presented medullary thyroid cancer in our cohort of incidentally identified carriers unaware of their *RET* status perhaps suggests that some early-stage disease found through screening thyroidectomy might not progress to clinically significant disease. The high prevalence of C-cell hyperplasia in both moderate-risk variant carriers and healthy individuals,^29^ coupled with the low medullary thyroid cancer risk we observed, may support this hypothesis. Further research is needed to understand this lower medullary thyroid cancer risk in incidentally identified cases and explore potential genetic modifiers of penetrance as previously reported in *BRCA*-related cancer and monogenic diabetes.^9,10,30^

Our study addresses the previously missing evidence on incidentally identified pathogenic *RET* variants. Current recommendations are based on the benefits and risks observed in clinically ascertained families due to lack of available data for large numbers of incidentally identified cases. We present the first comprehensive data in this context, including disease prevalence (approximately 1 in 2000 carriers), variant distribution (approximately 98% moderate risk), disease risk (2.2%-19.0% at age 75 years), and all-cause mortality in the absence of prophylactic thyroidectomy (no association). The surgical risks with prophylactic thyroidectomy, although low (<10% chance of long-term complications),^31^ coupled with lifelong hormone dependence affecting quality of life in up to 20% of cases may become particularly pertinent against the lower medullary thyroid cancer risk in incidentally identified moderate-risk *RET* variant carriers.^4,31,32^ Although variant reporting may continue and intervention choice remains personal, if one opts for biomarker-driven monitoring in this context, our data suggest that this will require sustained surveillance for a long period, given the low medullary thyroid cancer incidence (1 case per 2299 person-years) and low rate of pheochromocytoma.

Importantly our data suggest that medullary thyroid cancer onset in moderate-risk *RET* variant carriers occurs predominantly in adulthood in genotype-first and even in phenotype-first approaches (with 100% and 84% developing medullary thyroid cancer after age 30 years, respectively). This late-onset pattern may not meet the criteria for newborn genomic screening programs (genotype-first study design), which typically prioritize conditions in which the majority manifest disease before age 5 years.^8^

### Limitations

Our study has several limitations. Despite analyzing more than 500 000 individuals, we had limited numbers with *RET* pathogenic variants, particularly high-risk variants, highlighting the need for larger studies. We also lacked all known pathogenic moderate-risk variants, so it is possible that some carriers may have different risk. While appropriate for our research question, the UK Biobank cohort showed a healthy recruitment effect, as clearly observed in sex-specific cancers.^33^ This effect and the minimum recruitment age of 40 years may have depleted the cohort of high- and highest-risk variants and missed individuals with medullary thyroid cancer who died earlier. However, this potential depletion did not apply to incidentally identified *RET* variants in individuals older than 40 years and their follow-up data. We observed a similar age distribution between variant carriers and noncarriers and a similar prevalence of medullary thyroid cancer in our cohort compared with previous nationwide estimates (7.5 vs 3.8 per 100 000 individuals), suggesting that this issue might not have substantially influenced our findings.^28^ Our use of electronic health record information to define medullary thyroid cancer may have missed some individuals, but our broad inclusion criteria encompassing any thyroid cancer, thyroidectomy (regardless of indication), and self-reported data helped capture most. We do not have calcitonin measures in our study cohort, and incorporating this marker might provide more personalized risk estimates. We were unable to confirm the variant using a second DNA analysis method, although we mitigated against technical false-positive results through manual Integrative Genomics Viewer checking. Moreover, samples from 52 of 77 individuals (67.5%) with a pathogenic *RET* variant were subjected to Sanger sequencing at Geisinger (as part of MyCode Genome Screening and Counseling Program), and the presence of the variant was confirmed. The replication of findings across 2 different cohorts in 2 countries strengthens our conclusions, though due to all the limitations, our estimates may represent a lower bound of true population penetrance. While additional data on family members of clinically referred patients as a comparator group would have been useful, their absence did not influence our primary results. As our study focused on adult participants, future studies involving younger participants may be valuable to refine the estimates derived from current studies.

## Conclusions

This cohort study found that incidentally identified moderate-risk *RET* pathogenic variants are associated with substantially lower medullary thyroid cancer risk compared with clinically ascertained cases, with no excess mortality without intervention. This evidence addresses a critical knowledge gap, supporting informed clinical decision-making for individuals with incidentally identified *RET* variants.

## Notes

Supplement 1. **eTable 1.** Characteristics of Study Cohorts **eTable 2.** Summary of the Codes Used to Define Both the Strict and Permissive Definitions **eTable 3.** MEN2-Causing *RET* Pathogenic Variant Classification and Number of Individuals With Variant in Each Study Cohort **eTable 4.** Pathogenic *RET* Variant Carriers Are Not Enriched in Any Specific Ancestry **eTable 5.** Clinical Features of *RET* Pathogenic Variant Carriers With Any Thyroid Cancer, Including Medullary Thyroid Cancer or Thyroidectomy **eTable 6.** Cases of Medullary Thyroid Cancer in Study Cohort and by American Thyroid Association Pathogenic *RET* Variant Categories **eFigure 1.** Meta-Analysis of the Risks for Medullary Thyroid Cancers in *RET* Carriers **eFigure 2.** The Age-Related Risk of Medullary Thyroid Cancer in Individuals With *RET* Pathogenic Variant Ascertained Clinically and in a Clinically Unselected Population and Health System–Based Cohort With the Matched Variants

Supplement 2.
Data Sharing Statement
