# Error in Figure 1

**DOI:** 10.1001/jamanetworkopen.2025.28458

**Published:** 2025-08-01

In the Original Investigation titled “Medullary Thyroid Cancer Risk and Mortality in Carriers of Incidentally Identified MEN2A *RET* Variants,”^1^ published June 27, 2025, there were errors in the number at risk tables of panels B and C of Figure 1. These numbers should read as follows: Non-*RET* carriers, 383 745, 383 739, 383 579, 382 871, 334 354, and 142 850; *RET* carriers, 169, 169, 169, 167, 146, and 62. This article has been corrected.^1^
