# Supplementary material for: Medullary Thyroid Cancer Risk and Mortality in Carriers of Incidentally Identified MEN2A RET Variants
Source: JAMA Netw Open. 2025 Jun 27;8(6):e2517937. doi: 10.1001/jamanetworkopen.2025.17937 (PMC12205402; doi:10.1001/jamanetworkopen.2025.17937)
Supplement: Supplement 1. — eTable 1. Characteristics of Study Cohorts eTable 2. Summary of the Codes Used to Define Both the Strict and Permissive Definitions eTable 3. MEN2-Causing RET Pathogenic Variant Classification and Number of Individuals With Variant in Each Study Cohort eTable 4. Pathogenic RET Variant Carriers Are Not Enriched in Any Specific Ancestry eTable 5. Clinical Features of RET Pathogenic Variant Carriers With Any Thyroid Cancer, Including Medullary Thyroid Cancer or Thyroidectomy eTable 6. Cases of Medullary Thyroid Cancer in Study Cohort and by American Thyroid Association Pathogenic RET Variant Categories eFigure 1. Meta-Analysis of the Risks for Medullary Thyroid Cancers in RET Carriers eFigure 2. The Age-Related Risk of Medullary Thyroid Cancer in Individuals With RET Pathogenic Variant Ascertained Clinically and in a Clinically Unselected Population and Health System–Based Cohort With the Matched Variants [file jamanetwopen-e2517937-s001.pdf]

Supplemental Online Content

West CE, Mirshahi UL, Ruth KS, et al. Variant spectrum, medullary thyroid cancer risk, and mortality in carriers of MEN2A *RET* variants. *JAMA Netw Open*. 2025;8(6):2517937. doi:10.1001/jamanetworkopen.2025.17937

eTable 1. Characteristics of Study Cohorts

eTable 2. Summary of the Codes Used to Define Both the Strict and Permissive Definitions

eTable 3. MEN2-Causing *RET* Pathogenic Variant Classification and Number of Individuals With Variant in Each Study Cohort

eTable 4. Pathogenic *RET* Variant Carriers Are Not Enriched in Any Specific Ancestry

eTable 5. Clinical Features of *RET* Pathogenic Variant Carriers With Any Thyroid Cancer, Including Medullary Thyroid Cancer or Thyroidectomy

eTable 6. Cases of Medullary Thyroid Cancer in Study Cohort and by American Thyroid Association Pathogenic *RET* Variant Categories

eFigure 1. Meta-Analysis of the Risks for Medullary Thyroid Cancers in *RET* Carriers

eFigure 2. The Age-Related Risk of Medullary Thyroid Cancer in Individuals With *RET* Pathogenic Variant Ascertained Clinically and in a Clinically Unselected Population and Health System–Based Cohort With the Matched Variants

This supplemental material has been provided by the authors to give readers additional information about their work.

© 2025 West CE et al. *JAMA Network Open*.

eTable 1. Characteristics of Study Cohorts

	UK Biobank	Geisinger cohort	Exeter Clinical cohort
**N (unrelated)**	383,914	122,640	1078
**Cohort setting**	Population from the UK	Heath system based regional cohort, Pennsylvania, USA	Clinically referred cases from the UK for *RET* genetic testing for suspected MEN2
**Age at recruitment, y**	57.3 (8.11)	59.1 (18.0)	47.3 (18.0)
**Female Sex, n (%)**	198,442 (51.7)	73,602 (60.0)	592 (56.4)
**Genetic data**	Whole exome	Whole exome	Targeted gene panel or Sanger sequencing
**Phenotype**	Electronic health record – Hospital, GP, cancer registry, surgical records, self-report	Electronic health record - Hospital, GP, cancer registry, surgical records	Clinician reported

Data is given as n (%) for categorical variable and mean (SD) for the continuous variables

© 2025 West CE et al. *JAMA Network Open*.

eTable 2. Summary of the Codes Used to Define Both the Strict and Permissive Definitions

Definitions used in the study	Electronic health records codes (AND/OR) used in the definition (data combined from Self report, Hospital episode statistics or General practice records)
**1. Main definition: malignant thyroid cancer with medullary histology**	Thyroid Cancer: C73 (ICD10), 193/1939 (ICD9), 1065(Self-Reported), B53../BB9B./ByuB./ZV10y/X40Ia/44A2./X78cT/Xa98X/B9240/BB5fz/BB5f./BX78cV (GP records)<br>Histology in cancer registry: 8510 (Cancer Registry), X78cT (GP records)
**2. Broad definition: efinition 1 AND/OR any thyroid cancer or thyroidectomy for any indication**	Thyroid Cancer: C73 (ICD10), 193/1939 (ICD9), 1065(Self-Reported), B53../BB9B./ByuB./ZV10y/X40Ia/44A2./X78cT/Xa98X/B9240/BB5fz/BB5f./BX78cV (GP records)<br>Thyroidectomy: B081/B082/B083/B084/B085/B086/B088/B089 (OPCS4), 71100/71102/71103/71104/71105/71101/71106/7110y/7110z/7110./711../71130/7113z/7113y/Xa80R/Xa80S/XE0AE/X40GP/XM0my (GP records)
**3. Phaeochromocytoma and/or any adrenal tumours and/or adrenal surgery**	Phaeochromocytoma: 8700 (Cancer Registry) 1236 (Self-reported)<br>Adrenal Cancer: C749/D35.00/D35.01/D35.02/E27.5 (ICD10),227.0/194.0 (ICD9), 8370/3 (Cancer Registry), 1067/1233 (Self-reported)<br>Adrenal Surgery: B222/B223 (OPCS4), 1232 (Self-reported), ORD60540, ORD60650, ORD60545, 00866, A60650, 60545, 60650, A60540, 60540, 3206069901, 78001564119, 70060650, 77013

© 2025 West CE et al. *JAMA Network Open*.

eTable 3. MEN2-Causing *RET* Pathogenic Variant Classification and Number of Individuals With Variant in Each Study Cohort

HGVS Nomenclature cDNA	HGVS Nomenclature Protein	American Thyroid association classification	UK Biobank unrelated	MyCode cohort unrelated	Clinical Cohort unrelated	The American College of Medical Genetics and Genomics classification
**1597G>A**	Gly533Cys	Moderate	-	-	2	PS3 PP1 PM2 PP4
**1826G>A or<br>1826G>T**	Cys609Tyr or<br>Cys609Phe	Moderate	7	2	5	PP1 PS4 PM1 PM2 PP3 PP4 or<br>PP1 PM2 PM1 PS4 PP4 PP3
**1831T>C**	Cys611Arg	Moderate	-	-	1	PM5 PM2 PS3
**1852T>G**	Cys618Gly	Moderate	-	-	1	PM1 PS4 PM2 PP3 PP4
**1852T>C**	Cys618Arg	Moderate	-	-	6	PP1 PS4 PS3 PM1 PM2 PP4
**1853G>C**	Cys618Ser	Moderate	-	-	5	PP1 PS1 PM5 PM2 PP3 PS4
**1858T>G**	Cys620Gly	Moderate	-	2	1	PM5 PM2 PP4 PP3
**1858T>C**	Cys620Arg	Moderate	-	-	7	PS4 PP1 PS3 PM1 PM2 PP4
**1858T>A**	Cys620Ser	Moderate	-	-	3	PP1 PM1 PS4 PM2 PP3 PP4
**1859G>T**	Cys620Phe	Moderate	-	-	1	PM5 PM2 PS4 PP4 PP3
**1859G>A**	Cys620Tyr	Moderate	-	3	-	PM5 PM2 PS4 PP4 PP3
**1860C>G**	Cys620Trp	Moderate	-	1	-	PM5 PM2 PS4 PP4 PP3
**1996A>G**	Lys666Glu	Moderate	6	2	2	PS3 PM2 PP1 PP5
**1998G>C**	Lys666Asn	Moderate	7	1	-	PS1 PM2 PM5 PS3 PS4 PP4
**1998G>T**	Lys666Asn	Moderate	7	5	-	PS1 PM2 PM5 PS3 PS4 PP4
**2018A>C**	Glu673Ala	Moderate	-	-	1	PS3 PP1 PP4
**2304G>C or<br>2304G>T or<br>2370G>C or<br>1891G>T or<br>2711C>T or<br>2752A>G**	Glu768Asn or<br>Glu768Asp or<br>Leu790Phe or<br>Asp631Tyr or<br>Ser904Phe or<br>Met918Val	Moderate	11	2	-	PS3 PP1 PS4 PM2 PP4 or<br>PS3 PP1 PS4 PM2 PP4 or<br>PS1 PM2 PS4 PP1 PP3 PP4 or<br>PS3 PP1 PS4 PM2 PP4 or<br>PS3 PS4 PP1 PM2 PP4 or<br>PM5 PP1 PM2 PP4 PP3
**2370G>T**	Leu790Phe	Moderate	11	-	7	PS1 PM2 PS4 PP1 PP3 PP4
**2410G>A**	Val804Met	Moderate	95	30	14	PS3 PP1 PP4
**2410G>C**	Val804Leu	Moderate	15	-	-	PS1 PM5 PM2 PP4 PP3 PS3 PP1 PS4
**2671T>G**	Ser891Ala	Moderate	9	25	8	PS3 PS4 PP1 PP4
**1900T>C or<br>1900T>G or<br>1900T>A or<br>1901G>A or<br>1901G>T or<br>1902C>G or<br>2647_2648delinsTT**	Cys634Arg or<br>Cys634Gly or<br>Cys634Ser or<br>Cys634Tyr or<br>Cys634Phe or<br>Cys634Trp or<br>Ala883Phe	High	1	4	35	PS3 PS4 PP1 PM1 PM2 PP4<br>(same for all)
**2753T>C**	Met918Thr	Highest	-	-	18	PS3 PS2 PM6 PS4 PM2 PP4

*In line with UKB publication policy, n<5 was combined.

© 2025 West CE et al. *JAMA Network Open*.

eTable 4. Pathogenic *RET* variant carriers are not enriched in any specific ancestry.

The frequency of pathogenic *RET* carriers is stratified by ancestry in the UK Biobank (left) and MyCode (right) cohort, and the number of MTC and thyroid cancer based on the broad definition is shown. The frequency of pathogenic variants is largely similar between ancestries. The number of MTC and thyroid cancers shown are too rare for statistical analysis.

	UK Biobank				***Geisinger MyCode***			
Ancestry	**Total (%) individuals in UKBB population (n=383,914)**	**Frequency of pathogenic *RET* variant carrier in each ancestry (n=169)**	**Number of MTC in pathogenic *RET* carriers (n=3)**	**Number of cases with Broad definition in *RET* carriers (n=4)**	**Total (%) individuals in MyCode population (n=122,640)**	**Frequency of pathogenic *RET* variant carrier in each ancestry (n=77)**	**Number of MTC in pathogenic *RET* carriers (n=10)**	**Number of cases with Broad definition in *RET* carriers (n=13)**
EUR	352947 (91.9)	153/352,947 (0.04%)	3	4	111,824 (91.2)	74/111,824 (0.07%)	9	12
SAS	8823 (2.3)	2/8823 (0.02%)	0	0	459 (0.4)	0	0	0
AFR	6887 (1.8)	2/6887(0.03%)	0	0	4637 (3.8)	1/4637 (0.02%)	1	1
AMR	0	0	0	0	2110 (1.7)	1/2100 (0.05%)	0	0
Other & Unknown	15257 (4.0)	12/15257 (0.08%)	0	0	3610 (2.9)	1/3610 (0.03%)	0	0

© 2025 West CE et al. *JAMA Network Open*.

eTable 5. Clinical Features of *RET* Pathogenic Variant Carriers With Any Thyroid Cancer, Including Medullary Thyroid Cancer or Thyroidectomy

Individuals, n	Cohort	Ancestry	Variant	MTC, n	Thyroid cancer or Thyroidectomy	Indication for thyroidectomy
**6**	UK Biobank	EUR	Cys634Tyr/ Leu790Phe/ Val804Met	3/6	6/6	MTC -3, Pluriglandular neoplasm-1, Thyrotoxicosis -1, Non-toxic Multinodular goitre -1
**1**	Geisinger MyCode	EUR	Cys609Tyr	Yes	Yes	MTC
**1**	Geisinger MyCode	EUR	Cys620Gly	Yes	Yes	MTC
**1**	Geisinger MyCode	EUR	Cys620Gly	No	Yes	Prophylactic Thyroidectomy
**1**	Geisinger MyCode	EUR	Cys620Tyr	Yes	Yes	MTC
**1**	Geisinger MyCode	EUR	Cys620Tyr	Yes	Yes	MTC
**1**	Geisinger MyCode	EUR	Cys620Tyr	Yes	Yes	MTC
**1**	Geisinger MyCode	AFR	Cys634Arg	Yes	Yes	MTC
**1**	Geisinger MyCode	EUR	Cys634Phe	No	Yes	Unknown – Surgery not Performed at Geisinger
**1**	Geisinger MyCode	EUR	Val804Met	Yes	Yes	MTC
**1**	Geisinger MyCode	EUR	Val804Met	No	Yes	Hyperthyroidism
**1**	Geisinger MyCode	EUR	Ser891Ala	Yes	Yes	MTC
**1**	Geisinger MyCode	EUR	Ser891Ala	Yes	Yes	MTC
**1**	Geisinger MyCode	EUR	Ser891Ala	Yes	Yes	MTC

*In line with UKB publication policy, n<5 was combined.

© 2025 West CE et al. *JAMA Network Open*.

eTable 6. Cases of Medullary Thyroid Cancer in Study Cohort and by American Thyroid Association Pathogenic *RET* Variant Categories

Prevalent and incident cases for UK Biobank and prevalent cases for MyCode and clinical cohort.

	**UK Biobank**	**Geisinger MyCode**	**Clinical cohort**
Whole cohort	29/383914 (0.0075%) (7.5/100,000, [CI 5.06 - 10.8])	23/122640 (0.0187%) (18.7/100,000 [CI 11.9-28.1])	779/1078 (72.3%)
RET pathogenic variant carriers – ALL	3/169 (1.8% CI 0.4-5.1)	10/77 (12.9% CI 6.4-22.6)	96/117 (82.1% CI 73.9-88.5)
Highest risk	-	-	8/18 (44.4% CI 21.5-69.2)
High risk	1/1 (100% CI 2.5-100)	1/4 (25% CI 0.6-80.6)	29/35 (82.9% CI 66.4-93.4)
Moderate risk*	2/168 (1.2% CI 0.1-4.2)	9/73 (12.3% CI 5.8-22.1)	59/64 (92.2% CI 82.7-97.4)
p.(Val804Met)*	1/95 (1.1% CI 0.02-5.7)	1/30 (3.3% CI 0.1-17.2)	13/14 (92.9% CI 66.1-99.8)
Moderate risk excluding p.(Val804Met)*	1/73 (1.4% CI 0.03-7.4)	8/43 (18.6% CI 8.4-33.4)	46/50 (92.0% CI 82.8-99.9)
Moderate risk Extracellular variants*	0/8 (0%)	5/8 (62.5% CI 29.0-96.0)	29/30 (96.7% CI 82.8-99.9)

*MTC comparison in non p.(Val804Met) vs p.(Val804Met) moderate risk carriers in UK Biobank (Fisher's Exact test p=1.00), MTC comparison in intracellular vs extracellular moderate risk carriers in UK biobank (Fisher's Exact test p=1.00). MTC comparison in non p.(Val804Met) vs p.(Val804Met) moderate risk carriers in Geisinger cohort (Fisher's Exact test p=0.07), MTC comparison in intracellular vs extracellular moderate risk carriers in UK biobank (Fisher's Exact test p=0.004).

© 2025 West CE et al. *JAMA Network Open*.

eFigure 1. Meta-Analysis of the Risks for Medullary Thyroid Cancers in *RET* Carriers

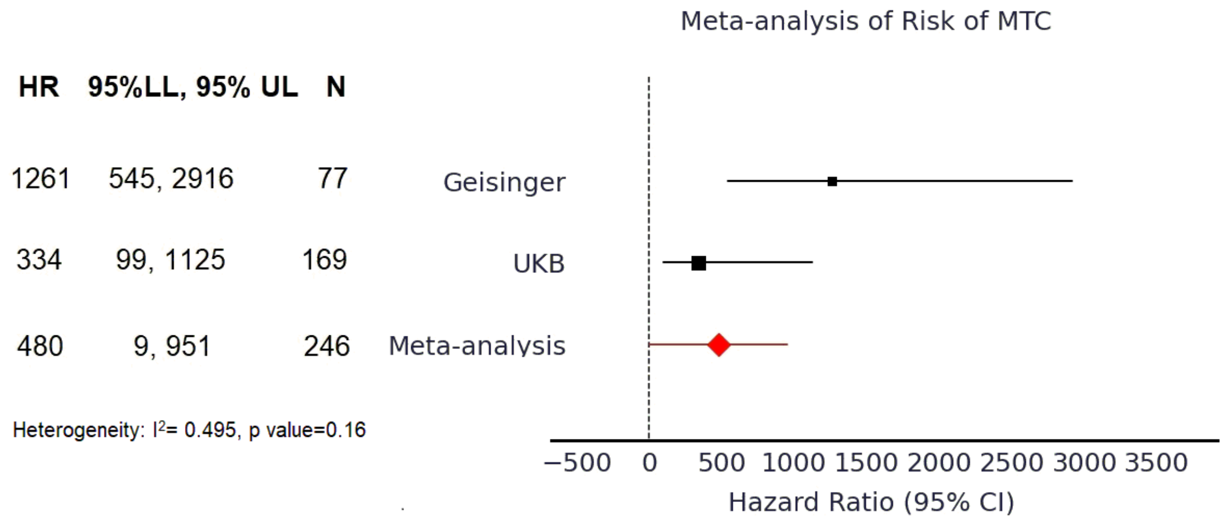

Forest plot showed the association of *RET* variant and MTC expressed as hazard ratios (HR) and 95%CI for the Geisinger MyCode and UKB cohorts. The pooled effect was calculated using the Cochran Q's fixed-effect model for dichotomous outcomes. The size of the data marker is proportional to the weight in the meta-analysis.

© 2025 West CE et al. *JAMA Network Open*.

A)

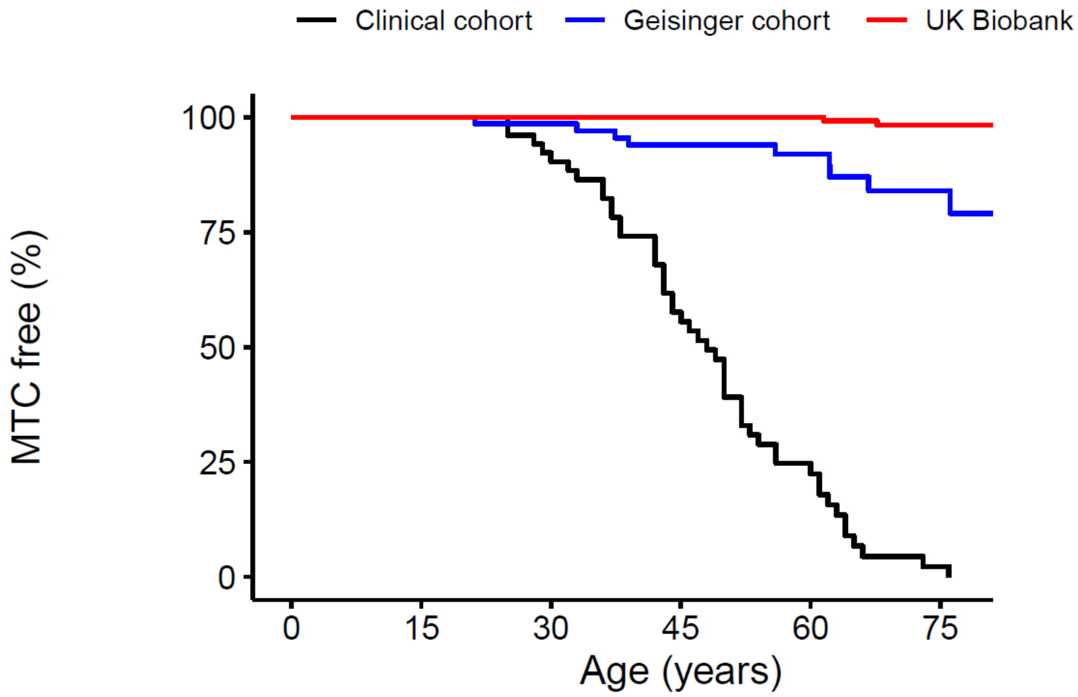

Clinical cohort	53	52	48	28	11	1
Geisinger cohort	73	73	67	56	42	18
UK Biobank	165	165	165	164	140	56

B)

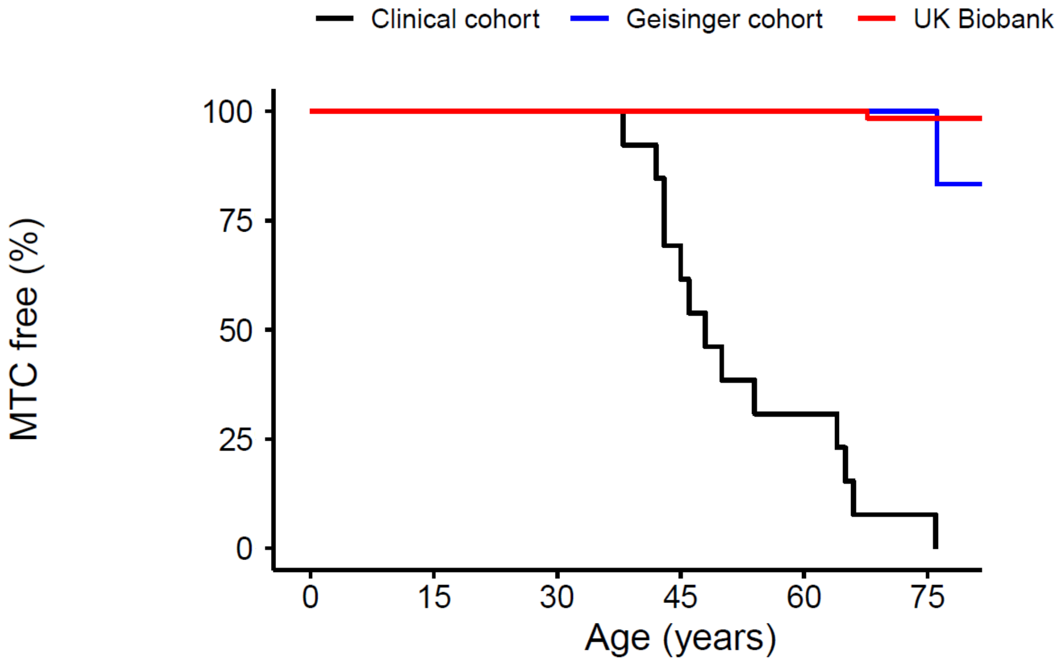

Clinical cohort	14	14	14	9	4	1
Geisinger cohort	30	30	27	22	14	7
UK Biobank	96	95	95	94	79	28

© 2025 West CE et al. *JAMA Network Open*.

C)

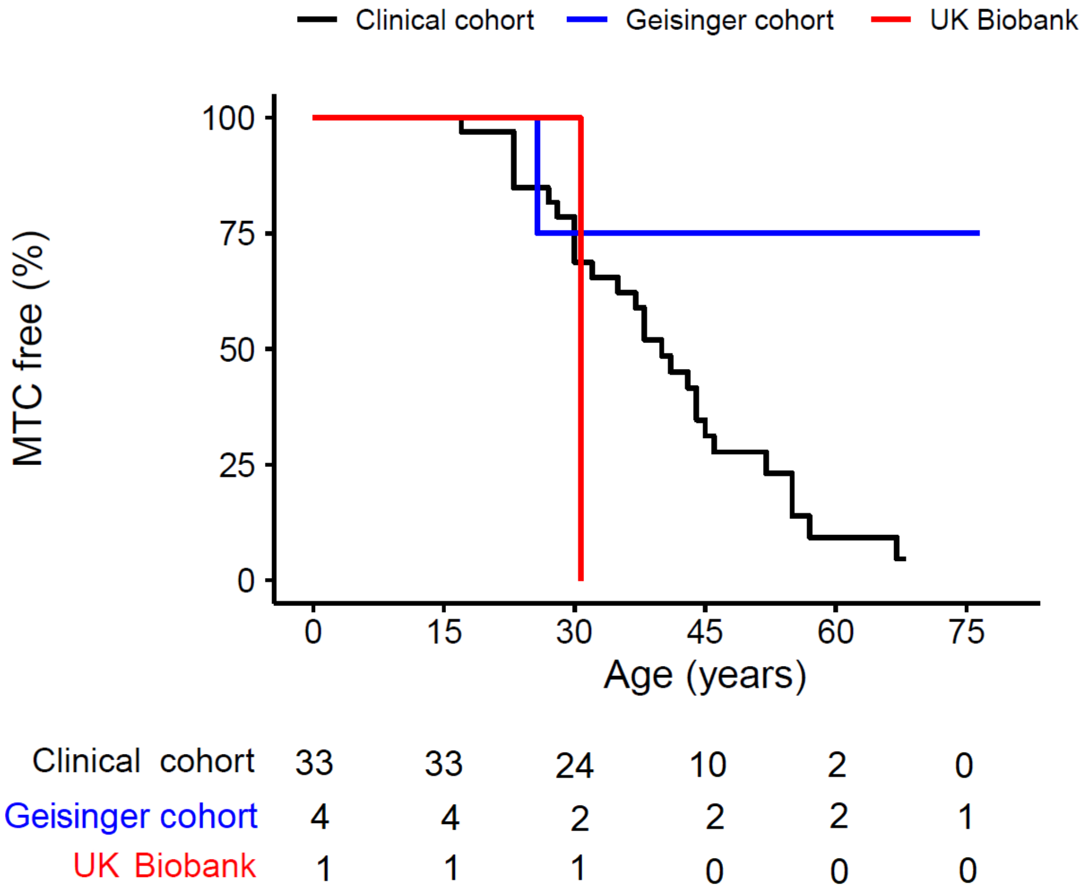

eFigure 2. The Age-Related Risk of Medullary Thyroid Cancer in Individuals With *RET* Pathogenic Variant Ascertained Clinically and in a Clinically Unselected Population and Health System–Based Cohort With the Matched Variants. A) Kaplan-Meier plot demonstrating the age-related penetrance of MTC for *RET* moderate risk pathogenic variant carriers identified from individuals referred to the Exeter genomic laboratory for genetic testing in routine clinical practice in the UK with suspected MEN2A and in unselected population cohort (UK Biobank) and health system-based cohort (Geisinger MyCode cohort), B) limiting to carriers with p.V804M in all three cohorts, C) carriers with high-risk variants in study cohorts.

© 2025 West CE et al. *JAMA Network Open*.
