# Supplementary material for: Medullary Thyroid Cancer Risk and Mortality in Carriers of Incidentally Identified MEN2A RET Variants
Source: JAMA Netw Open. 2025 Jun 27;8(6):e2517937. doi: 10.1001/jamanetworkopen.2025.17937 (PMC12205402; doi:10.1001/jamanetworkopen.2025.17937)
Supplement: Supplement 2. — Data Sharing Statement [file jamanetwopen-e2517937-s002.pdf]

Data Sharing Statement

West. Medullary Thyroid Cancer Risk and Mortality in Carriers of Incidentally Identified MEN2A RET Variants. *JAMA Netw Open*. Published June 27, 2025.
doi:10.1001/jamanetworkopen.2025.17937

Data
Data available: No

Additional Information
Explanation for why data not available: All the UK Biobank data used in this study is freely accessible from the UK Biobank https://www.ukbiobank.ac.uk. The pathogenic variants used in the study are already available in manuscripts. Data from the Geisinger MyCode cohort is available through the MyCode Community Initiative and the Geisinger-Regeneron DiscovEHR collaboration.
